# Co-Conserved MAPK Features Couple D-Domain Docking Groove to Distal Allosteric Sites via the C-Terminal Flanking Tail

**DOI:** 10.1371/journal.pone.0119636

**Published:** 2015-03-23

**Authors:** Tuan Nguyen, Zheng Ruan, Krishnadev Oruganty, Natarajan Kannan

**Affiliations:** 1 Department of Biochemistry & Molecular Biology, University of Georgia, Athens, Georgia, United States of America; 2 Institute of Bioinformatics, University of Georgia, Athens, Georgia, United States of America; University of Copenhagen, DENMARK

## Abstract

Mitogen activated protein kinases (MAPKs) form a closely related family of kinases that control critical pathways associated with cell growth and survival. Although MAPKs have been extensively characterized at the biochemical, cellular, and structural level, an integrated evolutionary understanding of how MAPKs differ from other closely related protein kinases is currently lacking. Here, we perform statistical sequence comparisons of MAPKs and related protein kinases to identify sequence and structural features associated with MAPK functional divergence. We show, for the first time, that virtually all MAPK-distinguishing sequence features, including an unappreciated short insert segment in the β4-β5 loop, physically couple distal functional sites in the kinase domain to the D-domain peptide docking groove via the C-terminal flanking tail (C-tail). The coupling mediated by MAPK-specific residues confers an allosteric regulatory mechanism unique to MAPKs. In particular, the regulatory αC-helix conformation is controlled by a MAPK-conserved salt bridge interaction between an arginine in the αC-helix and an acidic residue in the C-tail. The salt-bridge interaction is modulated in unique ways in individual sub-families to achieve regulatory specificity. Our study is consistent with a model in which the C-tail co-evolved with the D-domain docking site to allosterically control MAPK activity. Our study provides testable mechanistic hypotheses for biochemical characterization of MAPK-conserved residues and new avenues for the design of allosteric MAPK inhibitors.

## Introduction

Living cells use networks of mitogen activated protein kinases (MAPKs) to respond to diverse environmental signals. MAPKs include subfamilies such as the extracellular signal-regulated kinases (ERKs), p38 kinase, and c-Jun N-terminal kinase (JNK) that function as central nodes in signal transduction pathways associated with cell growth, differentiation, and apoptosis [1–3]. MAPKs belong to the CMGC group of kinases that include Cyclin-dependent kinases, Glycogen Synthase kinase and Casein kinase-2 [4]. MAPKs are related to CMGC and other protein kinases by virtue of the catalytic domain (also referred to as the kinase domain), which is activated and regulated by a diverse array of structural mechanisms [5,6]. Understanding how individual kinases are uniquely regulated in signaling pathways represents an important unmet challenge, which needs to be urgently addressed in order to selectively target abnormally regulated kinases in cancer and inflammatory disorders [7–10].

MAPKs share several regulatory features in common with other protein kinases. Regulation by activation loop phosphorylation (or A-loop, residues 174–183 in p38α) is one such feature [11,12]. Activation loop phosphorylation in MAPKs is mediated by the upstream MAP kinase kinase (MKK) [2,3,13]. Phosphorylation of the activation loop activates the kinase by reorienting key interactions in the ATP binding N-terminal lobe (N-lobe), including the conserved salt bridge between a lysine in the β3—strand and a glutamate in the regulatory αC-helix [1,2,5].

**Fig 1:**
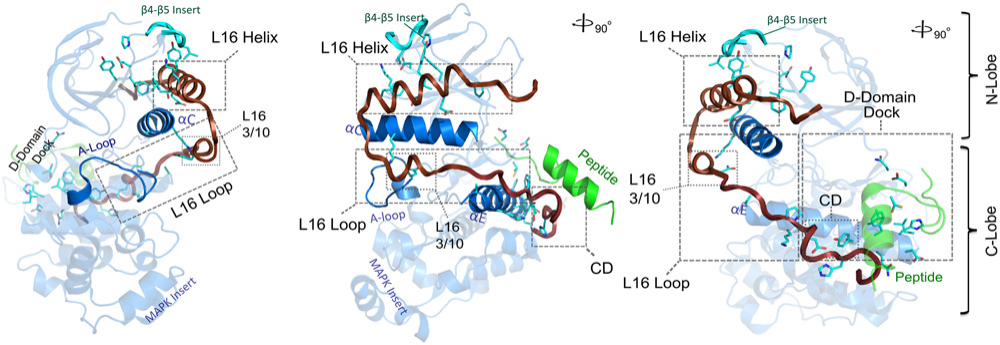
Structural delineation of MAPK specific residues (PDBID: 2OKR). Key structural regions being discussed in the text is highlighted, namely, the L16 helix and loop, D-domain/D-peptide docking site, CD (Common Docking site), αE and αC helices. Cyan sticks presentation denotes MAPK-specific residues identified via CHAIN (See Methods) and displayed in Fig. 2A alignment.

In addition to these conserved modes of regulation, MAPKs also display family-specific regulatory mechanisms to achieve specificity and temporal control. One mechanism through which MAPKs achieve specificity to their substrates and regulatory partners is through the D-domain/D-peptide docking site [14–16]. The D-domain docking site contains a hydrophobic docking groove flanked by the αD-αE linker (residues 119–134 in p38α), αE-helix (124–144), β7-β8 loop (159–163), and a common docking (CD) site (313–316), which is located in the C-tail [15,16] (Fig. 1). The D-domain docking site recognizes the consensus ψ_1–3_X_3–7_Φ-X-Φ substrate sequence motif, which is shared by diverse MAPK substrates (where ψ, X, Φ denote positively charged, intervening, and hydrophobic residues, respectively) [14–16]. Although first deemed as a static docking site to increase substrate binding affinity, numerous studies have presented evidence that the D-domain docking groove is a dynamic allosteric site that can bind to upstream effectors to either negatively or positively regulate protein kinase activity [14,16,17]. For example, peptide from the Ste5 scaffold protein binds to the D-domain docking groove of Fus3 MAPK, activating it by inducing long range conformational changes in the activation loop [18,19]. Structural studies of p38 revealed that TAB1 peptide binding to the D-domain docking region induces *cis*, MKK independent, phosphorylation of the activation loop [20]. Additionally, studies of c-Jun N-terminal (JNK) MAPK revealed JIP1 peptide-induced conformational changes in the active site via D-domain docking, resulting in catalytic inhibition [21,22]. Recent NMR studies support this view by showing allosteric activation of p38 MAPK upon D-domain peptide docking [17].

**Fig 2:**
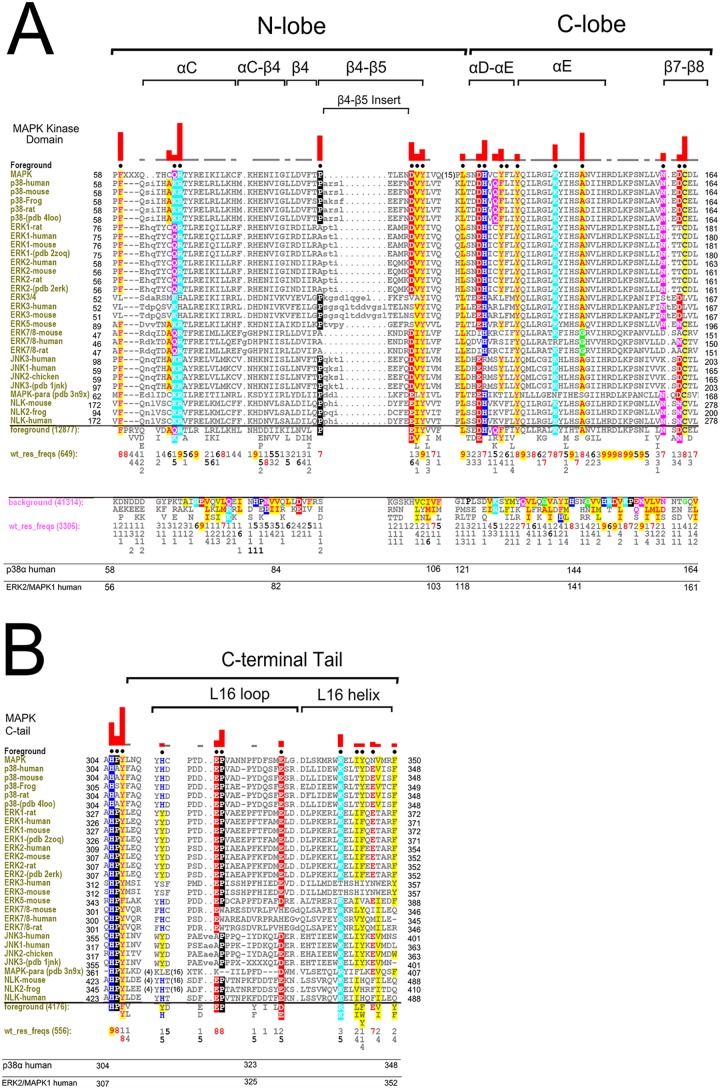
A and B) Selective constraints imposed upon MAPKs (foreground) that most contribute to sequence divergence from CMGC Kinases (background). Representative alignment of MAPKs is shown. Residues identified via CHAIN as distinctive of MAPKs are shown with black dots above each aligned column. The height of the red histograms above each corresponding column shows the degree of divergence of the MAPK foreground sequence from the CMGC counterparts (background). The residue frequency in the foreground is shown directly below the display alignment in positive integer tenths (i.e. 6 corresponds to 60–70% of weighted foreground sequences). The number of residues aligned in the foreground and weighted foreground frequency is shown near the “foreground” and wt_res_freqs designation, respectively. In particular, the weighted frequency was automatically done by the sampler to adjust to the overrepresentation of selected subfamilies in the alignment. The number of residues in the background and the weighted frequency is shown below in pink, respectively. Residues with strongest constraints are colored accordingly with residues of similar biochemical properties having similar color. Notable secondary structural locations are indicated above the histograms, and general numbering scheme is shown at the bottom with designated family numbering for reference. In Fig. 2B, the C-tail starts after the conserved HPY motif of the kinase domain.

In addition to the D-domain docking site, the sequence segment C-terminus of the kinase domain (L16 loop and L16 helix in Fig. 1 and Fig. 2B; and referred to as the C-tail) plays a critical role in MAPK activation and regulation. In p38α MAPK, for example, phosphorylation of a tyrosine (Y323) in the C-tail (L16 loop) regulates activity and downstream signaling in T-cells [23,24]. Likewise, mutations in the C-tail regulate MAPK activity [25] by inducing conformational changes in the kinase domain [26,27]. Furthermore, the Mxi2 isoform of p38 MAPK, which lacks the C-tail [28,29] displays low affinity for canonical p38α substrates and inhibitors, and deletion of the C-tail in p38α mimics the biochemical properties of Mxi2 [29]. More recent studies have highlighted the role of C-tail in MKK mediated interaction with ERK5 [30]. However, the residues and structural interactions that contribute to the unique modes of MAPK regulation by the C-tail are not fully understood.

Conformational control of catalytic activity by N and C-terminal segments flanking the kinase domain is a common mechanism of kinase regulation. For example, in receptor tyrosine kinase such as Ephrin Receptor Kinase (Eph), c-KIT, and FLT3, the N-terminal juxtamembrane segment plays an auto-inhibitory role by regulating the αC-helix and A-loop movement [31]. In ErbB kinases, on the other hand, the juxtamembrane segment plays an activating role [32]. Also, recent studies with Raf kinase have shown that residues in the N-terminal tail are required for catalytic activity [33]. In Ca^2+^/Calmodulin Dependent Protein Kinase, the C-terminal pseudosubstrate segment plays an auto-inhibitory role by blocking substrate binding [34]. In AGC and ErbB kinases, as well, the C-tail plays a regulatory role by interacting with key functional regions of the kinase domain [35–37].

Although MAPKs are well characterized signaling components that have been studied from a genomic perspective [38], the sequence and structural basis for MAPK evolutionary divergence is not fully appreciated. Our previous statistical sequence comparisons of CMGC kinases, to which MAPKs belong, revealed modular architecture of co-conserved motifs in the C-terminal lobe (C-lobe) that structurally link the conserved CMGC Insert (commonly referred to as MAPK Insert) to the peptide binding region [39]. Here, we build on these previous studies to provide a residue-level mapping of MAPK evolutionary divergence by comparing and contrasting MAPK sub-families with each other. We also quantify the evolutionary constraints distinguishing MAPKs from other CMGC kinases and present evidence for co-evolution between the C-tail and the kinase domain. In particular, we show, for the first time, that virtually all distinguishing MAPK residues, including an unappreciated MAPK-specific insert in the β4-β5 loop, couple the flanking C-terminal segment (C-tail) to the D-domain docking site and the regulatory αC-helix. This coupling appears to facilitate a unique mode of allosteric regulation in MAPKs involving the C-tail, D-domain docking groove, and MAPK-specific β4-β5 insert. Subfamily specific residues are built upon the core MAPK features to provide additional layers of control. Many distinctive subfamily sequence motifs, for example, appear to contribute to the positioning of the conserved acidic residue in the L16 3/10 helix to control catalytic activity. Our studies suggest a model for MAPK divergence in which the C-tail co-evolves with the D-domain docking groove to exert allosteric control of MAPK activity.

## Results

### Co-conserved Features Associated with MAP Kinase Domain Couple D-Domain Docking Site to the C-tail

To ascertain sequence and structural features that contribute to MAPK functional divergence, we used the Contrast Hierarchical Alignment and Interaction Network Analysis (CHAIN) program [40] to identify sequence constraints that most distinguish MAPK sequences (foreground) from related CMGC kinase sequences (background). Residues under strong evolutionary constraints are shown in Fig. 2A. These residues are generally highly conserved in MAPKs, but strikingly different in other CMGC sequences (See Method). In Fig. 2A, representative MAPK sequences from diverse organisms and subfamilies are used for display alignment. All MAPK sequences (12,877 sequences) correspond to the foreground, and all related CMGC kinase sequences outside MAPKs (41,314 sequences) correspond to the background. Residues that contribute most to MAPK sequence divergence, as quantified by the sampler (see Method), are shown in black dots above aligned columns with extent of divergence shown by the height of the histograms. These residues are generally well conserved within MAPKs but strikingly different in other CMGC kinases.

To perform a more exhaustive subfamily specific analysis within MAPKs, we used a more automated approach via the multi category Bayesian Partitioning with Pattern Selection (mcBPPS) procedure [41] (See Methods) to identify selective sequence constraints imposed upon MAPK sequences and subfamilies, both within the conserved kinase domain and the variable C-tail. We note that sequence constraints identified through both CHAIN and mcBPPS methods are in agreement and do not affect our final interpretations. In this analysis, we use p38α as the prototypical MAPK since it best conserves MAPK-specific features. The residues identified by CHAIN and mcBPPS as MAPK-specific include (in p38α human numbering scheme): F59, K66, R67, P92, D101, V102, Y103, L122, D125, H126, Q128, F129, L130, Y132, K139, A144, N159 and C162 (Fig. 2A). Of these, L122, D125, H126, Q128, F129, and L130 form the D-domain peptide docking interface. Others such as R67^p38α^ (R73^p38γ^) in the αC-helix coordinates with the phosphorylated residue in the activation loop [42].

Whereas the roles of some MAPK-specific residues have been noted in the context of D-domain-peptide docking and activation loop phosphorylation, the roles of many others are poorly understood. Residues of unknown function include (in p38α numbering): F59, K66, P92, D101, V102, Y103, Y132, K139, A144, N159 and C162. Additionally, our analysis revealed an unappreciated short insert segment in the β4-β5 loop that is present in many MAPKs (termed the MAPK β4-β5 insert, from A93^p38^ to L96^p38^) (Fig. 2A). The β4-β5 insert is distinct from the MAPK Insert in the C-lobe that is shared by other CMGC kinases [43]. Although highly dispersed in sequence, MAPK-conserved residues form clearly interpretable interactions: they spatially interact with the C-tail (L16 loop and helix) and form the D-domain docking site, from which the C-tail emanates. Thus, virtually all MAPK specific features couple the D-domain docking groove to distal regulatory sites in the N-lobe via the C-tail (Fig. 1).

### The C-tail is a Distinguishing Feature of MAPKs

The C-tail contains key residues and motifs that distinguish MAPKs from other protein kinases. The distinguishing residues in the C-tail include (in p38α numbering): H312, E317, P318, E328, K338, T341, Y342, E344, and F348 (Fig. 1 and 2B). These residues are unlikely to be involved in folding-related functions because the kinase domain adopts the protein kinase fold without the C-tail, as demonstrated by crystal structures of diverse protein kinases [44], and the crystal structure of ERK3 (MAPK) without the C-tail (PDBID: 2I6L, unpublished). Rather, the selective conservation of these residues across diverse MAPK subfamilies spanning diverse eukaryotic phyla suggests a role for these residues in MAPK-specific functions. To obtain insights into such functions, we analyzed the structural interactions associated with MAPK-conserved residues in crystal structures. We also performed molecular dynamics simulations to assess the roles of these residues in MAPK dynamics. Our discussions of MAPK-specific interactions below are organized into three regions based on co-conserved interactions between the kinase domain and the C-tail. These include: (i) the N-terminal end of the C-tail (i.e. L16 loop region) that interacts with the E-helix and the common docking (CD) site in the kinase domain, (ii) the L16 3/10 helix in the C-tail that interacts with the αC-helix, (iii) the generally hydrophobic interface of the L16 helix that docks to the αC-helix and the β4-β5 MAPK specific insert in the kinase domain (Fig. 1).

### Conserved Interactions between the αE-helix and the C-tail

**Fig 3:**
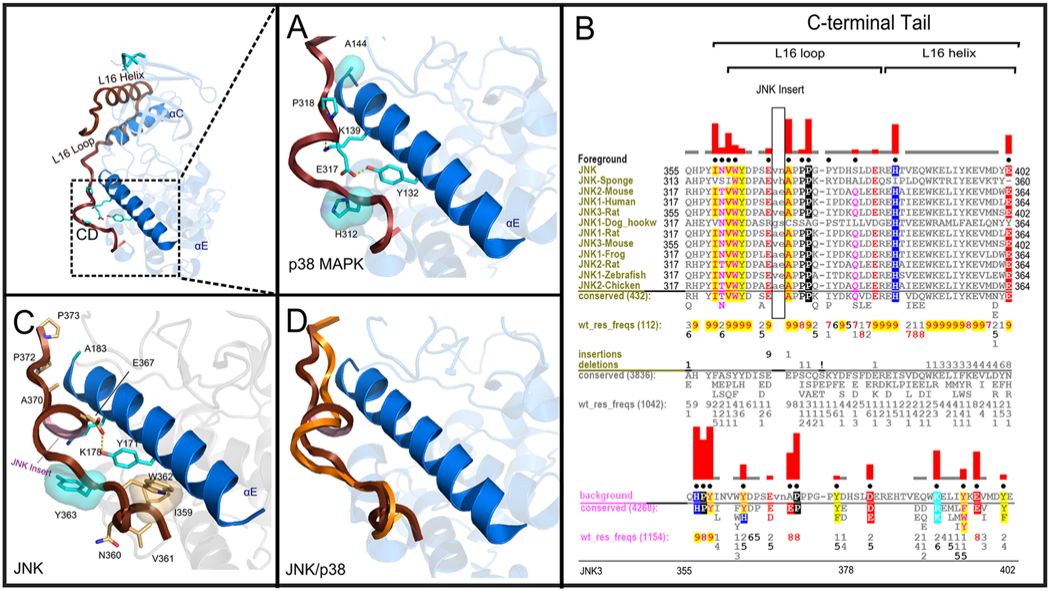
Structural location of conserved interactions between the αE-helix, the flanking C-tail, and the CD (Common Docking Site) region. A) Key conserved interactions in MAPKs, using a p38α as a representative model (PDBID: 4LOO), with cyan sticks representation denoting MAPK specific residues. Brown segment denotes the C-tail. B) Display set of sequence constraints imposed upon JNK C-terminal segment. General labelling schemes follow that of Fig. 2; the background corresponds to MAPK alignment while foreground corresponds to JNK alignment with number of sequences in alignment shown, respectively. The numbering schemes are shown at the bottom for each representative family. C) Divergent interaction of JNK in the analogous regions (PDBID: 1JNK) with JNK specific residues at the interface identified via the samplers. JNK-specific residues are shown in light orange, MAPK specific residues are shown in cyan, and JNK Insert is shown in light pink. D) Structural alignment of JNK’s C-tail (in brown) with a canonical p38α MAPK C-tail (in orange).

Conserved interactions tether the C-tail (L16 loop) to the αE-helix in the kinase domain. In particular, a lysine (K139^p38^) and tyrosine (Y132^p38^) in the αE-helix hydrogen bond to a glutamate in the L16 loop (E317^p38^ Fig. 3A), respectively. Additionally, a histidine (H312^p38^) (Fig. 3A) in the C-tail forms a CH-pi interaction with a hydrophobic residue in the αI helix (A304^p38^) (not shown). These tethering interactions are likely to play important regulatory roles because the tyrosine (Y132^p38^) is nitrated *in vitro*, and nitration of Y132^p38^ abolishes p38 catalytic activity [45–47]. Moreover, the αE-helix is part of a common docking site (CD), which interacts with many upstream proteins in MAPKs [15,16,21,22].

### JNK-Specific Variations and NLK Insert

While the interactions tethering the C-tail and αE-helix are generally highly conserved, several sub-family-specific variations are observed, providing insights into sub-family functional specialization. In C-Jun N-terminal Kinases (MAPK8/JNK), for example, the C-tail (L16 loop) is tethered to the αE-helix in a unique fashion through several sub-family-specific interactions. In particular, residues within a short insert segment (JNK Insert) and a JNK-specific INVW motif (residues 359–362 in Fig. 3B) in the C-tail mediate van der Waals and hydrogen bonding interactions with residues in the αE-helix [48] (Fig. 3C and 3D).

Sub-family specific variations in the L16 loop (Fig. 2B and S1 Fig.) are also observed in Nemo-like kinase (NLK), a highly divergent MAP kinase implicated in Wnt signalling pathway [3,49]. In particular, NLK is characterized by a 20 residue insert segment close to the CD site (Fig. 2B and S1 Fig.). The functional significance of the NLK-specific insert is not known, but the selective conservation of the insert proximal to the CD site suggests an important role in NLK functions.

### Conserved Interactions Coupling the MAPK β4-β5 Insert, αC-helix and α16 helix

**Fig 4:**
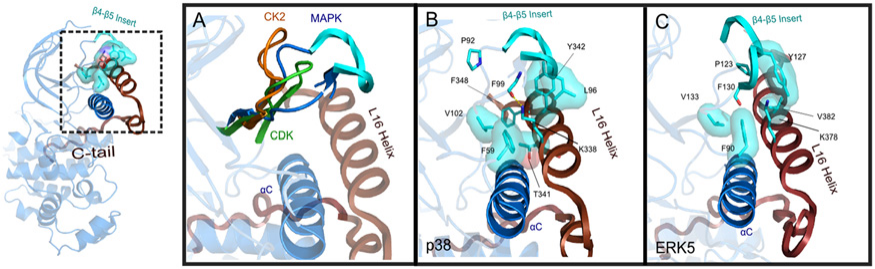
Interactions of MAPK β4-β5 Insert, αC-helix, and L16 helix. A) Structural alignment of MAPK β4-β5 loop with representative canonical CMGC kinase, where orange denotes Casen Kinase II (CK2) (PDBID: 1JWH), green denotes cyclin dependent kinase (CDK) (PDBID: 1QMZ), and blue denotes MAPK with insert colored in cyan with rectangle representation (PDBID: 4LOO). B) Conserved MAPK-specific interactions in p38α, with MAPK specific residues shown in cyan (PDBID: 4LOO). C) ERK5 variations in the equivalent region, where discussed interactions are shown (PDBID: 4IC7).

The conserved pocket along the αC-helix, termed the “αC patch”, is subject to allosteric control in diverse EPK families with numerous binding partners ranging from flanking segments in AGC kinases to cyclin in cyclin dependent kinase (CDKs) [32]. In MAPKs, the spatial vicinity of the “αC patch” consists of highly divergent features including the conserved β4-β5 Insert (Fig. 1A and Fig. 4A) and the KXXI/LF/YXEXXXF motif (Fig. 1B) in the C-tail. This region in MAPKs is characterized by unique hydrophobic interactions that couple the L16 helix, αC-helix and β4-β5 Insert (Fig. 4B). Together these interactions appear to uniquely tether the C-tail to the αC-helix and β4-β5 Insert in the kinase domain.

We also note that kinases outside of the MAPK family (S2 Fig.) share some similarities in the interactions associated with the αC patch. In particular, a tyrosine (Y574^PKC^) in the C-tail of Protein Kinase C (PKC) and a tryptophan (W342^C-raf^, W450^B-raf^, and W556^KSR1^) in the N-terminal flanking segment of Raf kinases (S2 Fig.) protrude into the hydrophobic pocket formed between the αC-helix and the β4 strand in a manner analogous to MAPK conserved phenylalanine (F348^p38^) in the L16 helix. Mutation of W450^B-raf^ has been shown to impair Raf catalytic activity [33], but the role of F348^p38^ in MAPK has not been explored.

### Non-canonical L16 Helix Interactions and β4-β5 Insert Segments in ERK3, ERK5 and Apicomplexan MAPK Orthologues

Sub-family-specific variations are observed in interactions coupling the L16 helix, β4-β5 Insert, and the αC-helix in MAPKs. For example, in ERK5, a MAPK subfamily involved in cell growth and neural differentiation [1,3], the tyrosine in the L16 helix (Y342^p38^) is replaced by a less bulky valine (V382^ERK5^) (Fig. 2B), which packs up against the bulky aromatic group of Y127^ERK5^ from the β4-β5 Insert (Fig. 4C). The β4-β5 Insert in ERK5 is repositioned relative to p38 (Fig. 4B and 4C). Consequently, K378^ERK5^ in the L16 helix does not hydrogen bond to the β4-β5 Insert (F130^ERK5^) (Fig. 4C). The length of β4-β5 Insert also varies in several subfamilies. In particular, ERK3/MAPK6 [50] contains an unusually long β4-β5 Insert (12 residue insert instead of a 3–4 residue insert in canonical MAPKs) (Fig. 2A). In the only solved structure of ERK3, the β4-β5 Insert is partially disordered (not shown) with portions of the C-tail (L16 loop and helix) truncated, which may contribute to the high B-factors of the αC-helix (PDBID: 2I6L). Notably, ERK3 diverges from the canonical MAPK residues in the C-tail, along with the unusually long insert. Specifically, in ERK3, the basic arginine/lysine in the L16 helix (K338^p38^/K378^ERK5^) is substituted to a threonine, and the histidine/tyrosine (H312^p38^) is substituted to a serine (Fig. 1B). In ERK7/8 (MAPK15), the β4-β5 Insert is not observed (Fig. 2A) and the MAPK-specific proline within the C-tail (P318^p38^) is substituted to a tryptophan (Fig. 2B).

Further structural analysis of Apicomplexan-specific MAPK orthologues revealed notable variations in the interactions between L16 helix, β4-β5 Insert and αC-helix. MAPKs in Plasmodium berghei (*P*. *berghei*), for example, conserve a distinctive helical extension that contributes to an extended interface between the β4-β5 Insert, αC-helix, and L16 helix (S3 Fig.). The helical extension appears to rearrange the canonical conformation of the β4-β5 Insert. Further functional implications of these variations are discussed in the Discussion section.

### Conserved Salt Bridge Interactions Coupling the L16 3/10 Helix and αC-helix

Precise positioning of the αC-helix is critical for kinase activation, and such is achieved through diverse mechanisms in EPKs. In MAPKs and related CMGC kinases, the αC-helix conformation is controlled, in part, through phosphorylation of a tyrosine and threonine residue (T175^p38^ and Y177^p38^) in the activation loop [1]. Phosphorylation of the activation loop results in positioning of the αC-helix in an active conformation by facilitating allosteric interactions between a positively charged residue (R70^p38^) in the αC-helix and the negatively charged phosphate group in the activation loop [42,51–53].

**Fig 5:**
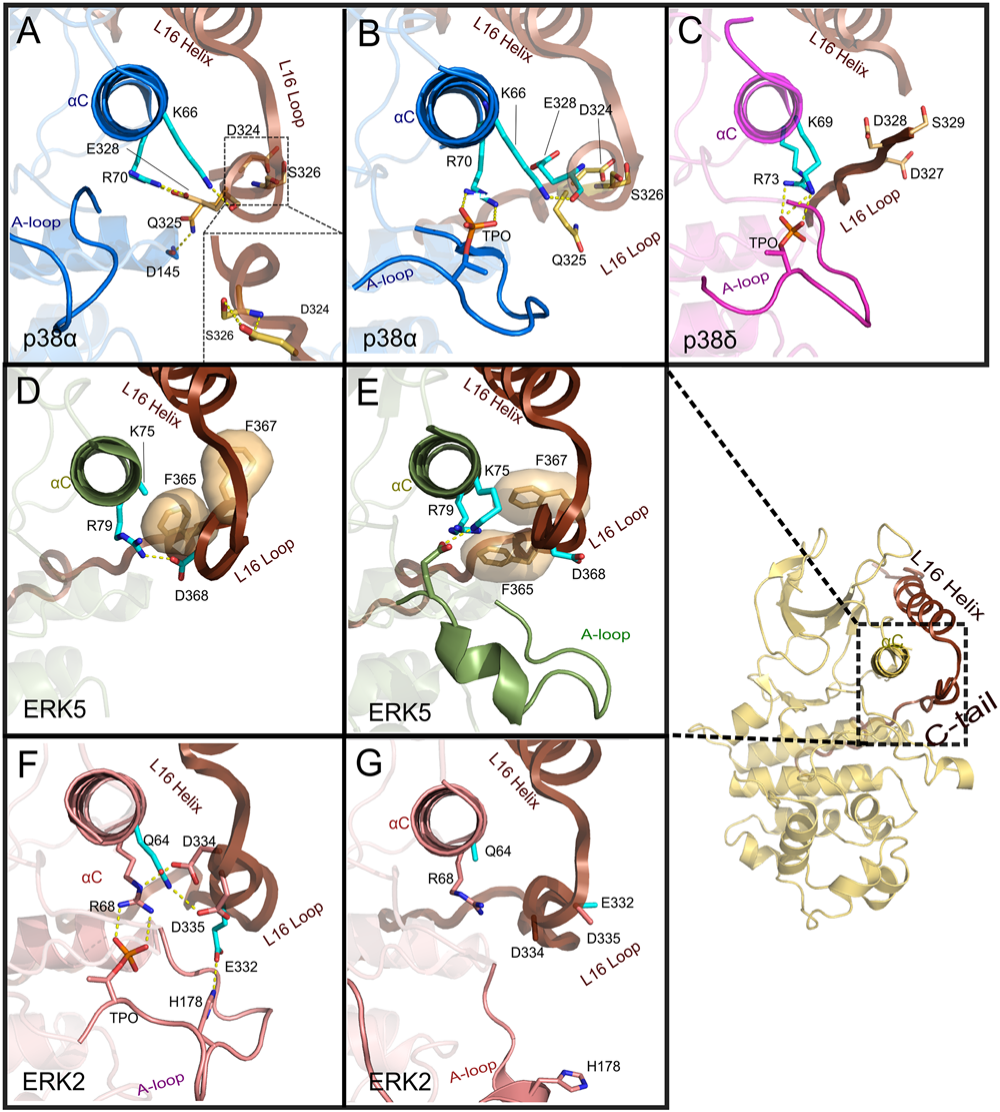
Interactions between the conserved acidic and associated residues in the L16 3/10 Helix. Residue coloring scheme is similar to Fig. 3 (i.e. cyan sticks representation denotes MAPK specific residues and light orange/tan sticks denotes subfamily (e.g. JNK) specific residues). A) Interactions of the MAPK specific salt bridge in the apo, unphosphorylated p38α (PDBID: 1P38). B) Interactions of the corresponding region in phosphorylated, apo form of p38α (PDBID: 3PY3). C) Interactions of the salt bridge in ATP analog bound, dually phosphorylated state in p38γ (PDBID: 1CM8). D) Interactions in apo, unphosphorylated ERK5 (PDBID: 4IC8). E) Interactions in ATP analogue bound, unphosphorylated ERK5 (PDBID: 4IC7). F) Interactions in phosphorylated, ATP bound ERK2 (PDBID: 2ERK). G) Disengagement of canonical salt bridge in apo ERK2 (PDBID: 4GSB), where the side chains of E332^ERK2^ and D334^ERK2^ are disordered.

MAPKs have diverged from other CMGC kinases by structurally coupling the allosteric interactions between the activation loop and the αC-helix with the L16 loop in the C-tail [26,27]. A notable structural feature near the L16 loop is the short and malleable 3/10 helix (termed L16 3/10 helix) first noted in ERK2 [53,54]. Our analysis of selective constraints imposed upon sequences in this region revealed a conserved acidic residue (E328^p38^) (Fig. 2B) in the 3/10 helix. Structurally, the glutamate in the L16 3/10 helix forms a salt bridge with the αC-helix arginine (R70^p38^) in MAPKs. In p38, the salt bridge between E328^p38^ and R70^p38^ is formed only when activation loop T175^p38^ is unphosphorylated (Fig. 5A). Upon phosphorylation of T175^p38^, the salt bridge is lost due to conformational changes in L16 3/10 helix and coordination of R70^p38^ with the activation loop phosphate (Fig. 5B and 5C).

**Fig 6:**
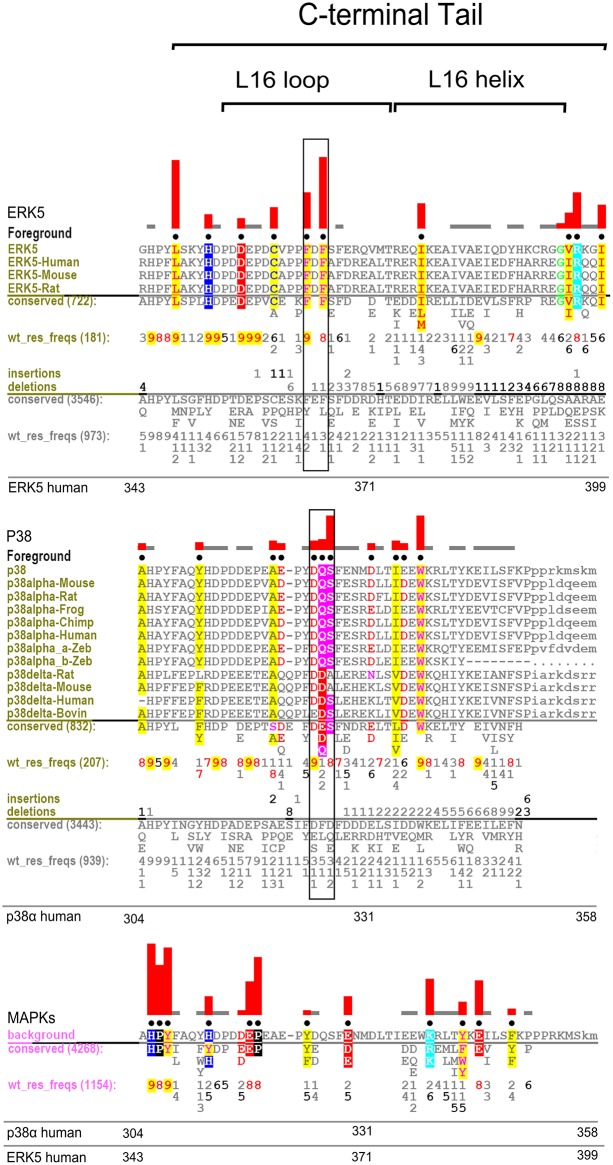
Display set showing selective sequence constraints imposed upon ERK5 and p38α flanking C-terminal segment. Top alignment shows ERK5 display (foreground) set with labels accordingly explained in Fig. 2; boxed sequence motifs denote discussed positions in the text. The middle alignment shows p38α (foreground) display set with labels similar to Fig. 2, and boxed sequence motifs denote discussed positions in the text. The background sequence at bottom corresponds to related MAPK sequences that belong to families outside of ERK5 and p38α, respectively.

These interactions are modulated in different ways in MAPK sub-families, presumably to facilitate unique modes of allosteric control. For example, in p38, the L16 3/10 helix adopts a unique conformation (mediated by the p38-specific DQ/DS motif in the C-tail (Fig. 6)), and the salt bridge interaction between E328^p38^ and R70^p38^ (Fig. 5A) is formed. Likewise, in ERK5, the unique L16 loop conformation (mediated by ERK5 specific FxF motif (Fig. 6)) alters the salt bridge interaction between R79^ERK5^ and D368^ERK5^ in the apo and ATP-bound forms (Fig. 5D and 5E).

ERK2 appears to diverge from other MAPKs in controlling the inter-lobe salt bridge via the L16 3/10 helix conformation. In the active state of ERK2, the MAPK-specific glutamate in the C-tail (E332^ERK2^, equivalent to E328^p38^) forms a polar interaction with H178^ERK2^ in the activation loop [53] instead of the canonical salt bridge interaction with R68^ERK2^ (R70^p38^) in the C-helix (Fig. 5F). Instead, R68^ERK2^, forms a partial salt bridge with an aspartate in the C-tail (D334^ERK2^) [53] (Fig. 5F). The loss of these interactions in the apo/inactive state of ERK2 suggests a role for these variations in ERK2-specific allosteric regulation (Fig. 5G).

### Molecular Dynamics Simulations

To further explore the role of the C-tail and D-domain peptide in MAPK structures and dynamics, we performed molecular dynamics (MD) simulations of MAPKs for two representative MAPK subfamilies, p38 and ERK, using the Gromacs package [55] (See Methods). In particular, we conducted MD simulations of p38 and ERK2 in apo and D-domain peptide bound forms to explore the role of the docking peptide in modulating MAPK structural dynamics. Our MD simulations were performed on four solved p38 and ERK2 constructs resolved in crystal structures (residues 5–351 in p38α; PDBID: 1P38 [56] and 4LOO [20] and residues 9–356 in ERK2; PDBID: 4GSB [57] and 3O71 [58]). We further performed MD simulations of p38 and ERK2 without the C-tail (residues 9–306 in ERK2, and residues 5–307 in p38, which partially mimics the α2 isoform [28,29]). Below, we present our main results in two sections: 1) dynamics of MAPKs in different states of activation, and 2) atomic level descriptions of key interactions during the simulations.

### Dynamics of MAPKs in Apo, D-Domain Peptide Bound and Truncated Form

**Fig 7:**
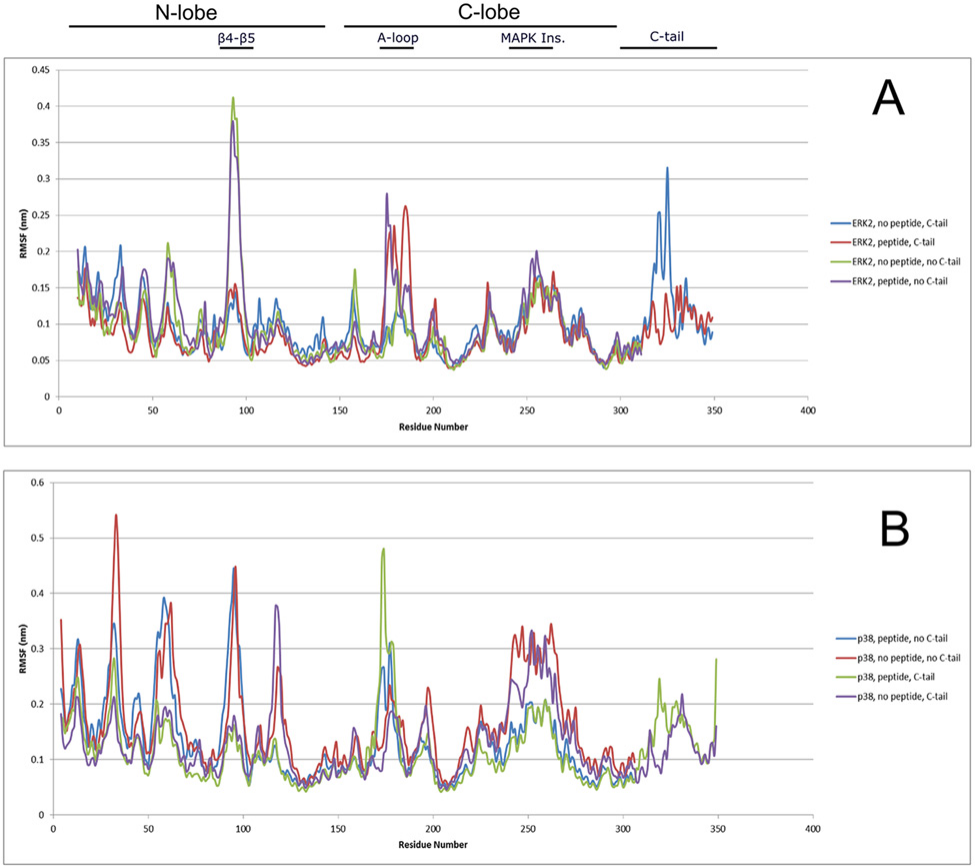
Root Means Square Fluctuation (RMSF) plot of A) ERK2 and B) P38α.

To explore the stability and how thermal fluctuations of MAPKs are modulated via the C-tail and D-domain peptide binding, we analyzed the root-mean-square deviations (RMSD) and root-mean-square position fluctuation (RMSF) of each MD trajectory. We note that both p38 and ERK2 structures remained stable during all simulations, particularly for the kinase core without the C-tail, as shown via RSMD plots (S4 and S5 Figs.). The largest differences in thermal fluctuations among the structures during simulations are observed in the β4-β5 region (residues ~90–100) of the N-lobe, the A-loop (residues ~170–180), the MAPK Insert (residues ~240–260), and the L16 loop of the C-tail (residues ~310–330), as shown via RMSF plot (Fig. 7).

**Fig 8:**
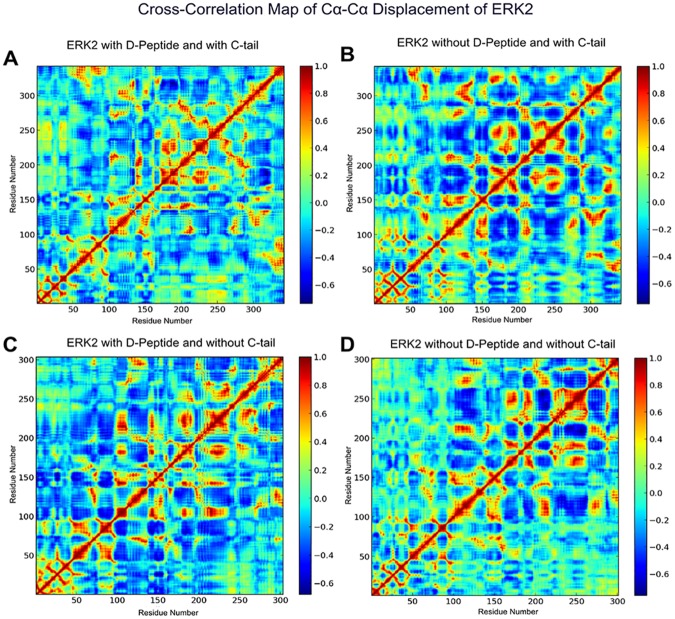
Cross correlation map of Cα-Cα displacement of ERK2 in A) D-Domain/D-Peptide Bound Form with C-tail, B) D-Domain Peptide Unbound (Apo) Form with C-tail, C) D-Domain Peptide Bound Form without C-tail, and D) D-domain Peptide Unbound (Apo) Form without C-tail.

In ERK2, the β4-β5 region displays enhanced thermal fluctuations in the kinase core without the C-tail (Fig. 7A). Indeed, C-tail truncation appears to increase anti-correlated motions between the N-and C-lobes (residues ~5–100 and ~150–250) in apo form (Fig. 8A and 8C), and decrease inter-lobe motion in the D-domain peptide bound form (Fig. 8B and 8D).

**Fig 9:**
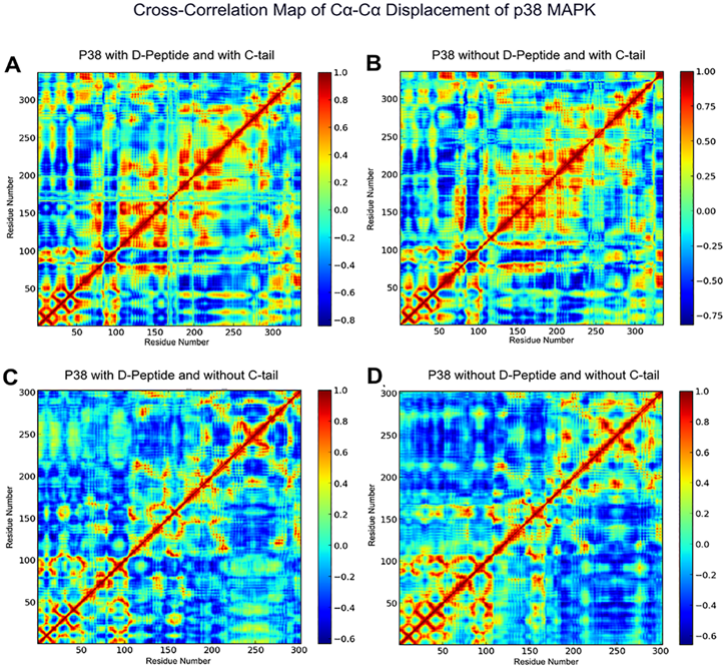
Cross correlation map of Cα-Cα displacement of p38α in A) D-Domain/D-Peptide Bound Form with C-tail, B) D-Domain Peptide Unbound (Apo) Form with C-tail, C) D-Domain Peptide Bound Form without C-tail, and D) D-domain Peptide Unbound (Apo) Form without C-tail.

In contrast to full-length p38, the kinase core of p38 without the C-tail displays enhanced thermal fluctuations in the N-lobe, suggesting that the N-lobe of p38 is intrinsically more flexible (Fig. 7B). The MAPK Insert in p38 displays reduced thermal fluctuation upon D-domain peptide binding, presumably due to the extended peptide segment interacting with the C-lobe [59] (Fig. 7B). Furthermore, the correlated dynamics of C-lobe (residues ~110–240) fluctuations appear to be significantly reduced upon truncation of the C-tail in both D-domain peptide bound and apo form (Fig. 9A,B and 9C,D).

In ERK2 and p38, both the presence of the C-tail and D-domain peptide binding appear to contribute, in an additive fashion, to enhanced thermal motions of the A-loop (residues ~170–180), a key regulatory component in EPKs [5] (Fig. 7A and 7B). This observation is consistent with the more correlated motion of the A-loop with the kinase domain upon D-domain peptide binding in p38 (Fig. 9A and 9B), as shown previously in JNK [60]. Furthermore, the dynamics of the L16 loop is also altered upon D-domain peptide binding (Fig. 7A-B), suggesting a role for the C-tail in MAPK allostery.

### Stability of C-tail and Kinase Domain Interactions

To understand how the C-tail contributes to structural regulation of MAPKs at the atomic level, we analyzed the hydrogen bonding patterns of key MAPK specific residues tethering the C-tail to the kinase domain.

As previously discussed, our sequence and structural analysis of MAPKs revealed a conserved salt bridge interaction between an acidic residue (E328^p38^) in the C-tail and an arginine (R70^p38^) in the regulatory αC-helix that is known to interact with a phosphorylated residue in the active state (Fig. 5). In our MD simulations of p38, the salt bridge interaction is stable in both apo and D-domain peptide bound form (>90% occupancy) (S1 Table). Furthermore, the capping interaction between a MAPK-specific lysine (K66^p38^) in the αC-helix and a glutamate (E328^p38^) in the C-tail (Fig. 5A and 5B) is malleable in both simulations (>60% occupancy). This observation is consistent with the conformational malleability of the L16 3/10 helix in different states of MAPK activation.

In ERK2 simulations, the αC-helix arginine (R68^ERK2^/R70^p38^) forms a stable salt bridge with D330^ERK2^ in the C-tail in the apo state (>99% occupancy). Interestingly, D330^ERK2^ is among the most distinguishing sequence positions in the ERK1/ERK2 subfamily (S6 Fig.). In the D-domain peptide bound form, R68^ERK2^ forms a less stable salt bridge (~65% occupancy) with D334^ERK2^ (S1 Table). Thus, ERK2 appears to have adopted unique variations in its C-tail to modulate αC-helix conformation.

## Discussion

The hinge-like inter-lobe dynamics and the precise positioning of the αC-helix and the activation loop are key steps in the catalytic cycle of protein kinases. MAPKs appear to have evolved a unique C-tail to couple these events to the latent allostery of the D-domain docking site via distinct interactions in the C-tail and the β4-β5 Insert. Indeed, the distinctive arrangement of residues involved in these interactions is highly conserved in MAPKs across diverse organisms, and many of which diverge from the corresponding position in other CMGC kinase. We propose that the unique interactions mediated by the MAPK C-tail provides a structural framework for allosteric coupling between peptide binding at the D-domain docking groove and catalytic control via αC-helix positioning and inter-lobe motion.

MAPK’s unique C-tail may be viewed as a *cis*-regulatory module that enables new modes of allosteric regulations through sequence and structural variations in the C-tail, such as regulation through C-tail Y323^p38^ phosphorylation in p38α MAPK [23,27]. Consistent with this notion, studies concerning the Mxi2 isoform of p38α suggest that alternatively spliced C-tail may be a mechanism to evolve new substrate specificity [28,29]. In addition, Hydrogen Exchange/Mass Spectrometry (HX/MS) studies revealed correlated conformational change of the flanking C-tail upon D-peptide docking [61]. Furthermore, NMR studies also support this model where docking of the allosteric TAB1 peptide to the D-domain binding site induces large scale conformational change in the N-lobe and the flanking C-terminal segment, facilitating activation loop phosphorylation [20]. Lastly, a previous study comparing the conformational states of JNK MAPK crystal structures across diverse space groups revealed inter-lobe conformational changes and rotation of the αC-helix upon binding of peptide activator to the D-domain docking site [21].

In addition to regulatory roles, the C-tail also provides an extended structural framework that enables evolution of MAPK functional diversity. Indeed, many MAPK subfamilies adopt unique interactions along the flexible L16 loop, such as p38α-specific phosphorylation of Y323, which plays unique physiological roles in T-cell signal transduction [23,24]. Furthermore, the C-tail may be alternatively spliced such as the Mxi2 isoform of p38 that displays different biochemical properties toward substrates [28,29] without the flanking C-tail. On the other hand, the hydrophobic region near the L16 helix in ERK2 may serve as a putative dimerization site that facilitates ERK2 nuclear translocation [62]. Our analysis of sequence constraints imposed upon JNK revealed a unique INVW motif and a conserved insert in the C-tail that contributes to structural rearrangement of the CD site, which likely explains JNK’s substrate specificity (Fig. 3B, C, and D). Likewise, the 20 residue insert in Nemo-like kinases (NLK) (Fig. 2B and S1 Fig.) near the CD site may serve as a unique docking site for regulatory proteins and substrates.

Structural comparisons of divergent sequence motifs in the L16 loop suggest variations in the salt-bridge mediated regulation of C-helix conformation. Indeed, the malleable nature of the salt bridge that is lost upon activation loop phosphorylation and ATP binding suggest a mechanistic model wherein the conserved acidic residue in the L16 3/10 helix functions as an electrostatic clamp by clamping the αC-helix arginine (R70^p38^ and R79^ERK5^) away from the phosphothreonine in the activation loop (Fig. 5). The clamp appears to be facilitated via sequence motifs unique to ERK5 and p38 near the malleable L16 3/10 helix. The proposed model of the electrostatic clamping offers a structural explanation for mutations in disease states, such as D324^p38^ in the DQ/DS motif, which is mutated to alanine in uterine corpus endometrioid carcinoma (COSMIC: COSM1078469; see also http://vulcan.cs.uga.edu/prokino/resource/Human_P38A). Through the proposed model, we predict that D324A mutation disrupts capping interactions in L16 3/10 helix, thereby loosening the electrostatic clamp. Our analysis of the L16 3/10 helix clamping interactions in ERK2 suggests that the clamp may play an activating role by ordering activation loop conformation.

Examination of MAPK dynamics via MD simulations also supports a regulatory role for the C-tail and dynamic control of catalytic activity (Fig. 7). Furthermore, D-domain peptide binding in both ERK1 and p38α induces altered thermal fluctuation in key structural regions, such as the L16 loop, the A-loop, and C-lobe region near the MAPK Insert. These events may contribute to tight allosteric regulation of MAPKs upon substrate binding and activation loop phosphorylation.

Previous analyses to characterize sequence features contributing to functional divergence of major EPK groups illustrate common themes of insert segments and co-conserved residues. The CMGC kinase, to which MAPKs belong, conserves an exaggerated CMGC insert (commonly referred to as MAPK Insert in literature) along with a CMGC specific network of contiguous residues that structurally couple the CMGC insert to the P+1 pocket [37,43]. Furthermore, both AGC and many tyrosine kinases adopt highly divergent interactions that structurally couple the *cis*-regulatory flanking segments to the αC-helix [36]. Indeed, MAPKs appear to display these two common themes of structural evolution. MAPK’s divergent sequence motifs form unique interactions with the C-tail, which emanates from the D-domain docking groove to interact with the “αC patch” via the L16 helix and the MAPK specific β4-β5 Insert. Altogether, these unique MAPK features may act in conjunction with other catalytic components to facilitate the switching between active and inactive conformations.

Regulation of αC-helix conformation by flanking segments appears to be a common theme in protein kinase regulation. Our analysis provides a concerted model of MAPK mechanistic divergence and evidence for co-evolution between the D-domain docking site, the C-tail, and the β4-β5 Insert for tight regulation of MAPK activity. A greater understanding of the flanking segment regulation within the context MAPK and EPKs will provide new avenues to dissect the functional components of these signaling systems. Thus, future studies that shed light on mechanistic connections and co-evolution between flanking segments and kinase domains will hold important implications for drug design and protein engineering.

## Methods

### Identification of MAPK Specific Constraints

EPK sequences from major taxonomic groups and families were aligned using the MAPGAPS program [63] using curated profiles of major EPK groups and families [44,64,65]. The C- tail sequence profile was curated using Fammer tool [64] and formatted for CHAIN analysis using biocma (https://github.com/etal/biocma). The selective constrains imposed on MAPK sequences was identified using CHAIN, which partitions 54,191 aligned protein kinase sequences (main set), into two divergent categories: the foreground, which contains MAPKs, and background, which contains non-MAPK CMGCs. Furthermore, the sampler identifies columns in the alignment that best distinguishes the foreground from the background. These correspond to sequence constraints unique to MAPKs, where the extent to which these sequence positions contributed to MAPK-divergence is quantified via a ball-in-urn statistical model [40] and qualitatively displayed by the height of histograms above the aligned columns (Fig. 2). Such co-conserved sequence patterns have previously been noted to mediate specific modes of allosteric communication network [35–37,66] and form protein-protein interaction surfaces [37,39,67]. Detailed description of CHAIN can be found in [40] and [68]. In addition to CHAIN, we also used mcBPPS, which performs multiple foreground-background comparisons simultaneously [41] to identify sequence patterns distinguishing MAPKs from CMGC kinases and patterns distinguishing the C-tail of MAPK-subfamilies (e.g. p38, ERK5, JNK etc) from each other. In brief, mcBPPS sampler uses the consensus query/seed alignment, usually the protein family of interest, and the hyperpartition tree of all EPKs to optimally partition the main master alignment into multiple hierarchical categories based on pattern selection [41].

### Structural Analysis of MAPK-Specific Interactions

Crystal structures of MAPKs were downloaded from the Protein Databank (PDB) (http://www.rcsb.org/). Electrostatic interactions and van der Waal interactions were calculated via CHAIN suite [40]. Described structural interactions were visualized using PyMol (http://www.pymol.org/). The following structures were used for our figures (denoted by PDBIDs): P38: 1P38 [56], 2OKR [69], 1WFC [70], 4LOO [59], 3PY3 [71], 1CM8 [42]. ERK2: 2ERK [53], 4H3Q [72], 4GSB [57]. ERK3: 2I6L (unpublished). ERK5: 4IC7 [30], 4IC8 [30], 4B99 [73]. JNK: 1JNK [74], 4H3B [75]. *P*. *berghei* MAPK orthologue: 3N9X (unpublished). CK2: 1JWH [76]. CDK2: 1QMZ [51]. B-RAF: 1UWH [77]. PKC: 4DC2 [78].

### Molecular Dynamics Simulations

All molecular dynamics (MDs) were performed using Gromacs version 4.6.4 [55]. For the MAPK simulations, PDBs 4LOO and 1P38 of p38, and 3O71 and 4GSB of ERK2 were taken as the starting structures after modeling the missing residues using Modloop [79]. For each MAPK subfamily (i.e. p38 and ERK2), a total of four MD simulations were performed: 1) D-domain peptide bound with C-tail (residues 5–351 in p38α and residues 9–356 in ERK2), 2) apo (no D-domain peptide) with C-tail, 3) D-domain peptide bound without C-tail (residues 9–306 in ERK2, and residues 5–307 in p38), and 4) apo (no D-domain peptide) without C-tail. In both p38 and ERK2, we used physiological D-domain peptide resolved in crystal structures: TAB1 for p38 [20], and DCC peptide for ERK2 [58].

The structures were prepared using the Gromacs program in a dodecahedron periodic box with each side at least 1nm away from the protein. The proteins were parameterized using AMBER99SB-ILDN force field and TIP3P water model was used. Long-range electrostatics were computed with PME method with a cutoff of 10 nm. Energy minimization using steepest descent and conjugate gradients was carried out before each MD run till change in potential energy became negligible with maximum force on any atom less than 100 kJ mol-1 nm-1. A time step of 2 fs was used for the equilibration and production runs and bond lengths and bond angles were constrained using a fourth order LINCS algorithm. Temperature equilibration was done in an NVT ensemble for 500 pico seconds at a temperature of 35°C with a Berendsen thermostat. Pressure and density equilibration was done for 1 nano second using Berendsen thermostat and Berendson barostat. For production runs, Parinello-Rahman barostat and Nose-Hoover thermostat were used. Production runs were 200ns for 4LOO and 1P38,the rest of the simulations (i.e. 4GSB, 3O71, and C-tail truncated forms) was run for 100 ns with frames written every 4 pico seconds. Comparisons of Cα- Cα correlation matrices between simulations were performed at the first 100ns of each simulation. Analysis of MD trajectories (i.e. hydrogen bonding and RMSF etc.) were done using packages in Gromacs suite, and cross correlation matrix of Cα- Cα displacement was generated using Prody [80]. In our MD analysis, we removed the extreme regions of the C-tail (residues 349–353 in 4LOO, residues 349–354 in 1P38 and residues 349–356 in 4GSB, residues 349–354 in 3O71, respectively), since inclusion of these flexible regions biases the calculations of cross correlation matrix towards the local tail motions instead of collective global motion.

## Supporting Information

S1 Fig Nemo-like Ser/Thr Kinase (NLK) CHAIN C-tail alignment. Labeling schemes follow that of Fig. 2 and Fig. 6, with the NLK Insert shown in the alignment. Background sequence alignment at bottom corresponds to general MAPK which don’t belong to NLK (Group-1-background). MAPK-conserved sequence motifs are shown as reference at bottom.
(PNG)

S2 Fig Structural resemblance of conserved interactions adjacent to the αC-β4 Pocket via exploiting the different flanking segments. A) MAPK’s C-tail (PDBID:4LOO). B) B-raf’s N-terminal Tail (PDBID: 1UWH). C) PKC’s C-tail (PDBID:4DC2).
(PNG)

S3 Fig MAPK orthologue variation in *P*. *berghei*. A) and B) *P*. *berghei* MAPK orthologue variations shown with corresponding MAPK specific features in cyan and non-MAPK specific residues in non-cyan color (PDBID:3N9X).
(PNG)

S4 Fig Root Mean Square Deviation (RMSD) plot of p38α MAPK with respect to superimposed starting crystal structures during the MD simulations. (PNG)

S5 Fig Root Mean Square Deviation (RMSD) Plot of ERK2 with respect to superimposed starting crystal structures during the MD simulations. (PNG)

S6 Fig ERK1/ERK2 C-tail alignment labeling schemes follow that of Fig. 2 and Fig. 6. The display set shows representative ERK2 C-tail sequences. The sequence columns highlighted in black dots show the positions that most contributed to ERK1/ERK2’s shared sequence features that contributed to both families’ divergence. The highlighted column (D330^ERK2^) denotes the referred position discussed in the text in MD simulations section. Structural interaction of D330^ERK2^ observed in MD simulations is shown in the structural figure beside the alignment (PDBID:4GSB).
(PNG)

S1 Table Occupancy during the MD simulation of key discussed interactions in the text. (PDF)
