# Supplementary figures and images for: Co-Conserved MAPK Features Couple D-Domain Docking Groove to Distal Allosteric Sites via the C-Terminal Flanking Tail
Source: PLoS One. 2015 Mar 23;10(3):e0119636. doi: 10.1371/journal.pone.0119636 (PMC4370755; doi:10.1371/journal.pone.0119636)

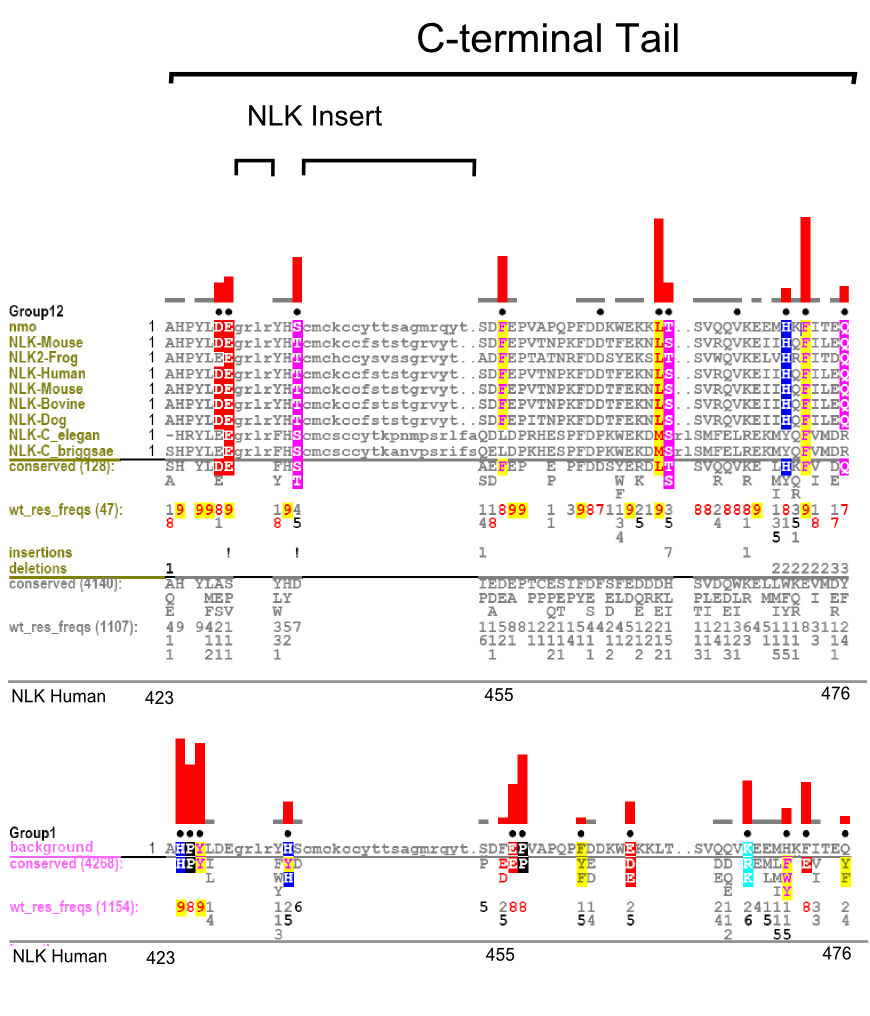

Supplement: S1 Fig — Labeling schemes follow that of Fig. 2 and Fig. 6, with the NLK Insert shown in the alignment. Background sequence alignment at bottom corresponds to general MAPK which don’t belong to NLK (Group-1-background). MAPK-conserved sequence motifs are shown as reference at bottom. (PNG) [file pone.0119636.s001.png]

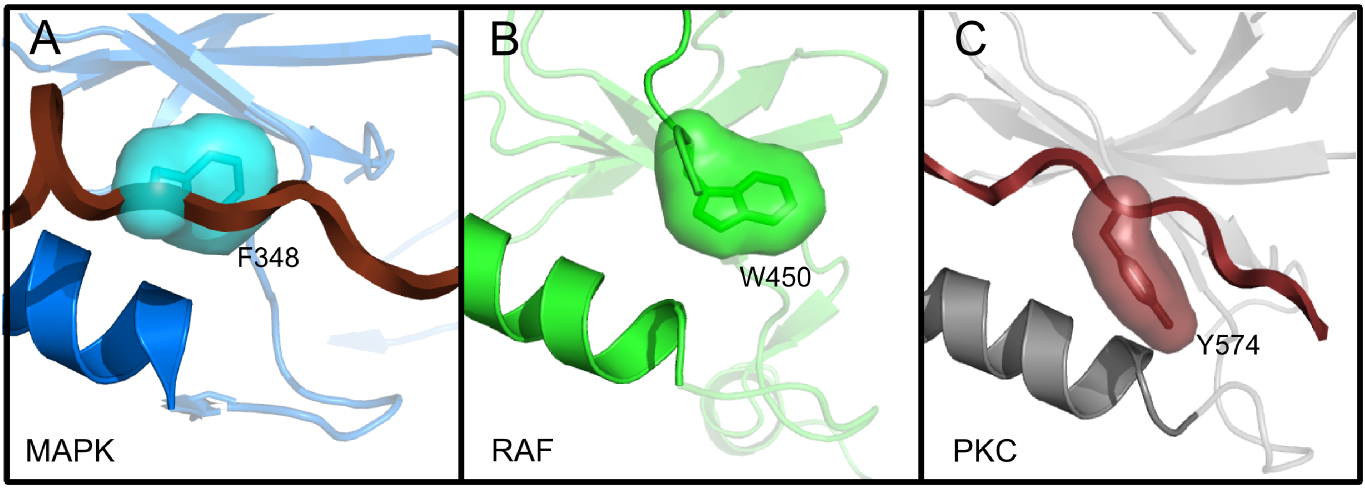

Supplement: S2 Fig — A) MAPK’s C-tail (PDBID:4LOO). B) B-raf’s N-terminal Tail (PDBID: 1UWH). C) PKC’s C-tail (PDBID:4DC2). (PNG) [file pone.0119636.s002.png]

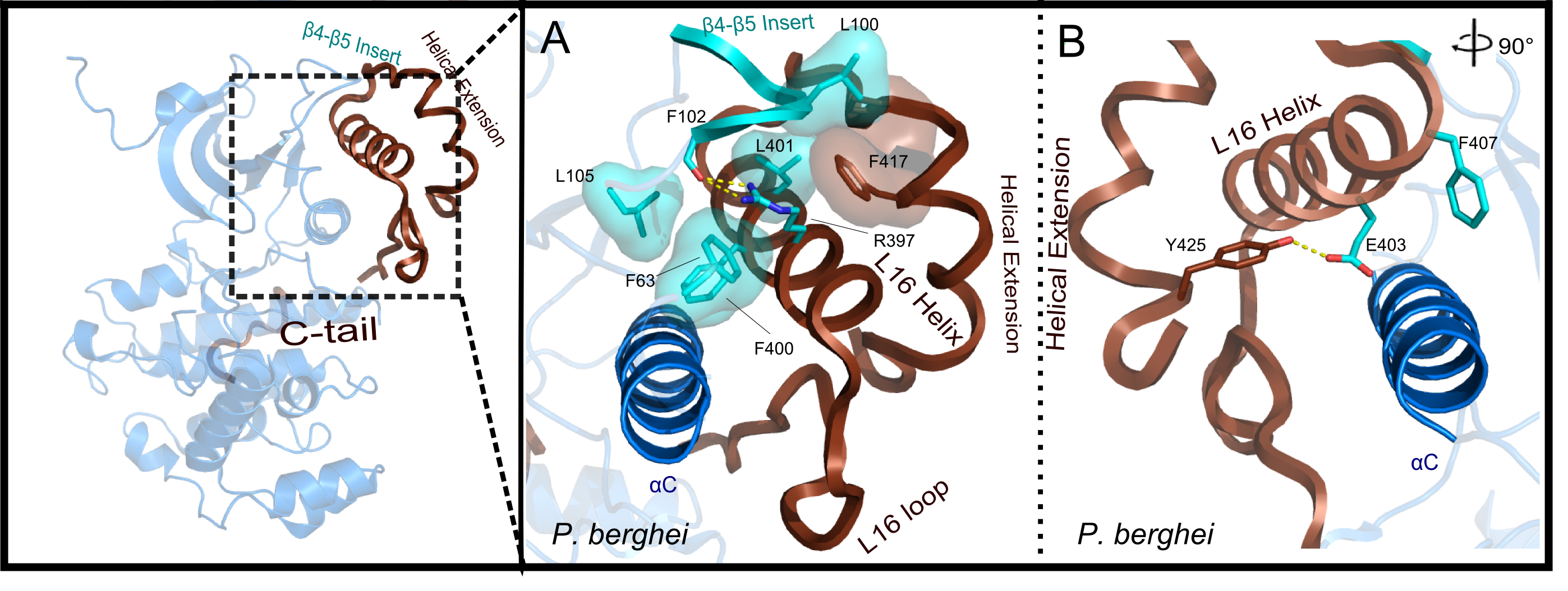

Supplement: S3 Fig — A) and B) P. berghei MAPK orthologue variations shown with corresponding MAPK specific features in cyan and non-MAPK specific residues in non-cyan color (PDBID:3N9X). (PNG) [file pone.0119636.s003.png]

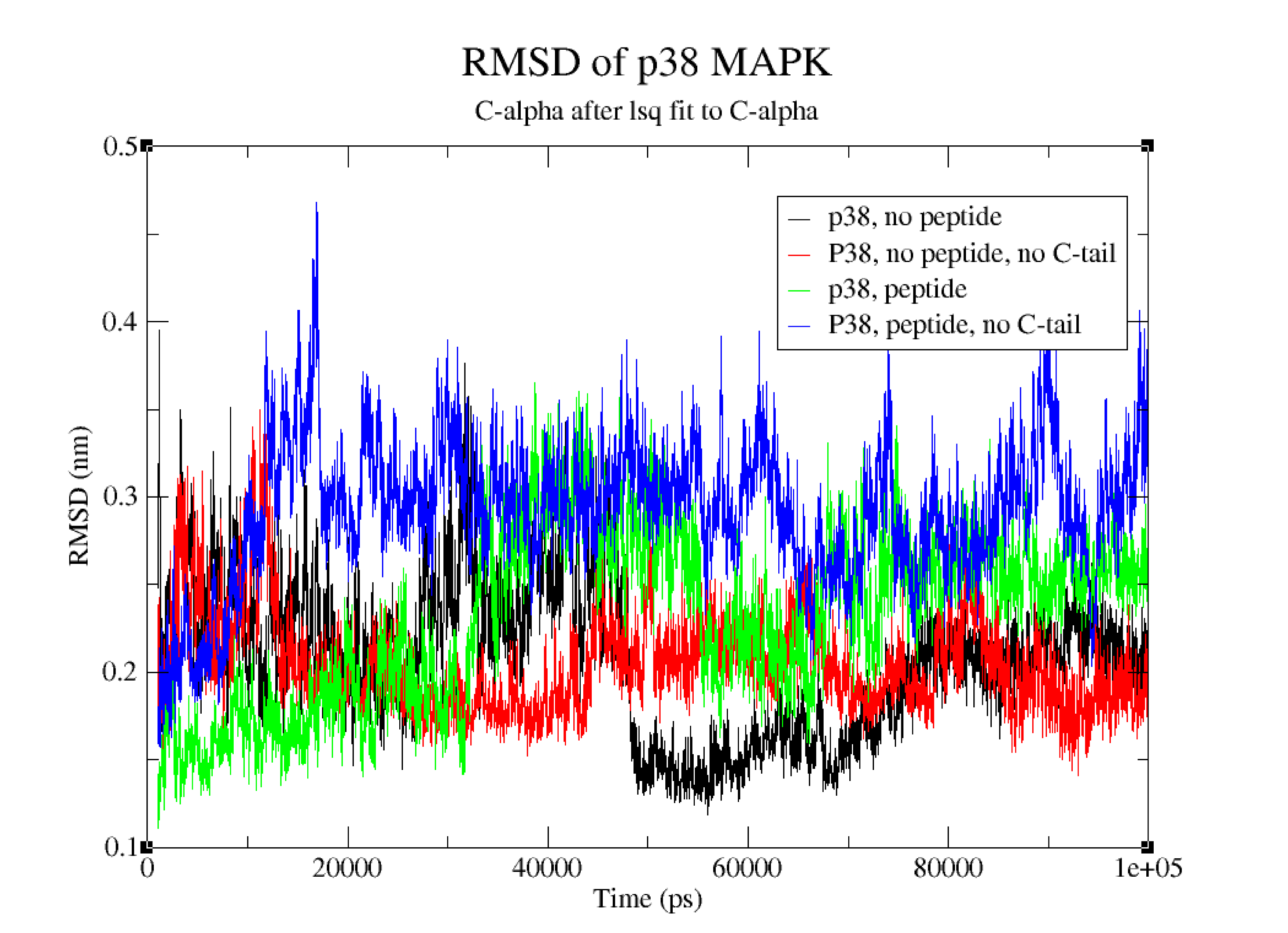

Supplement: S4 Fig — (PNG) [file pone.0119636.s004.png]

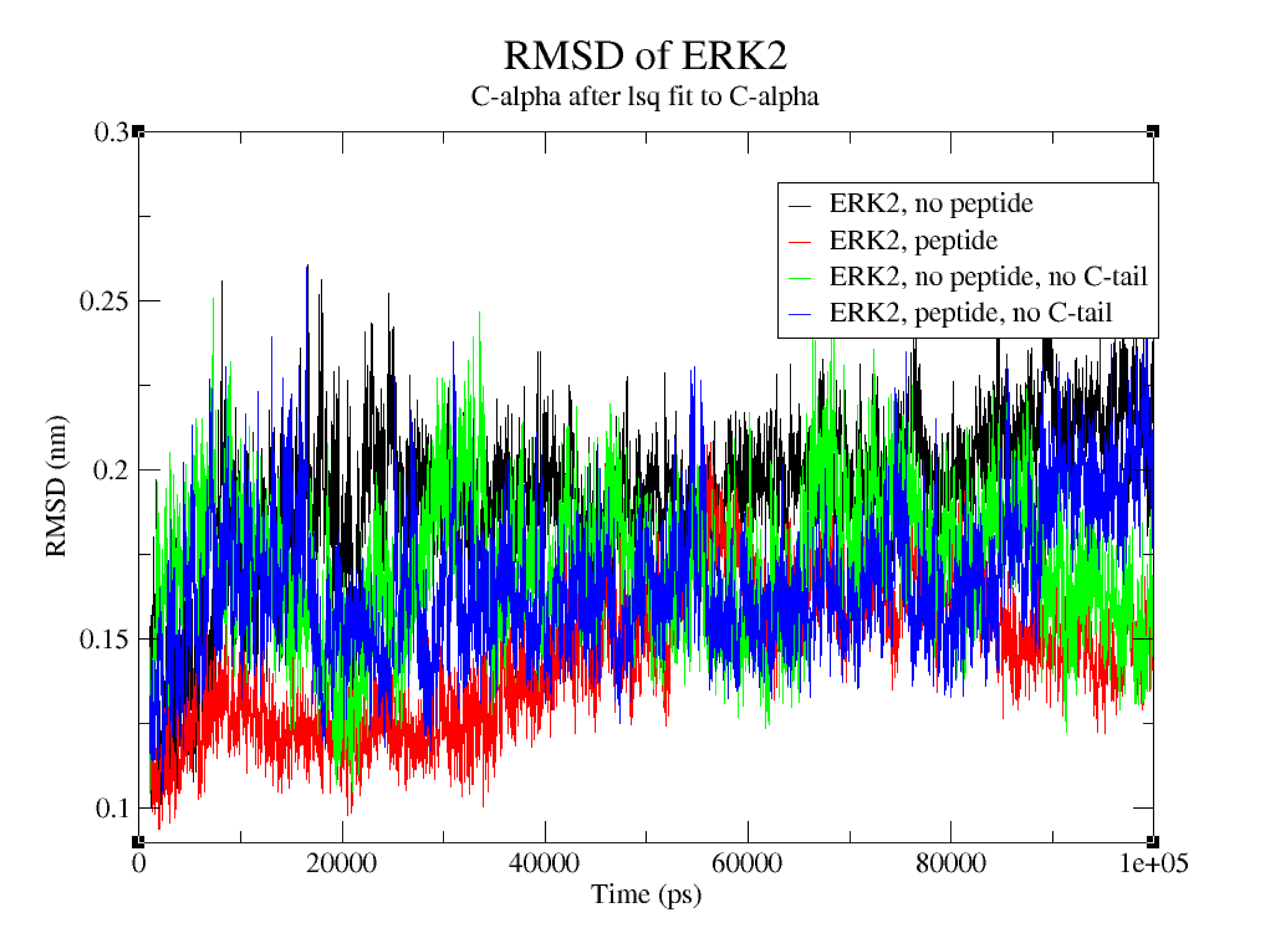

Supplement: S5 Fig — (PNG) [file pone.0119636.s005.png]

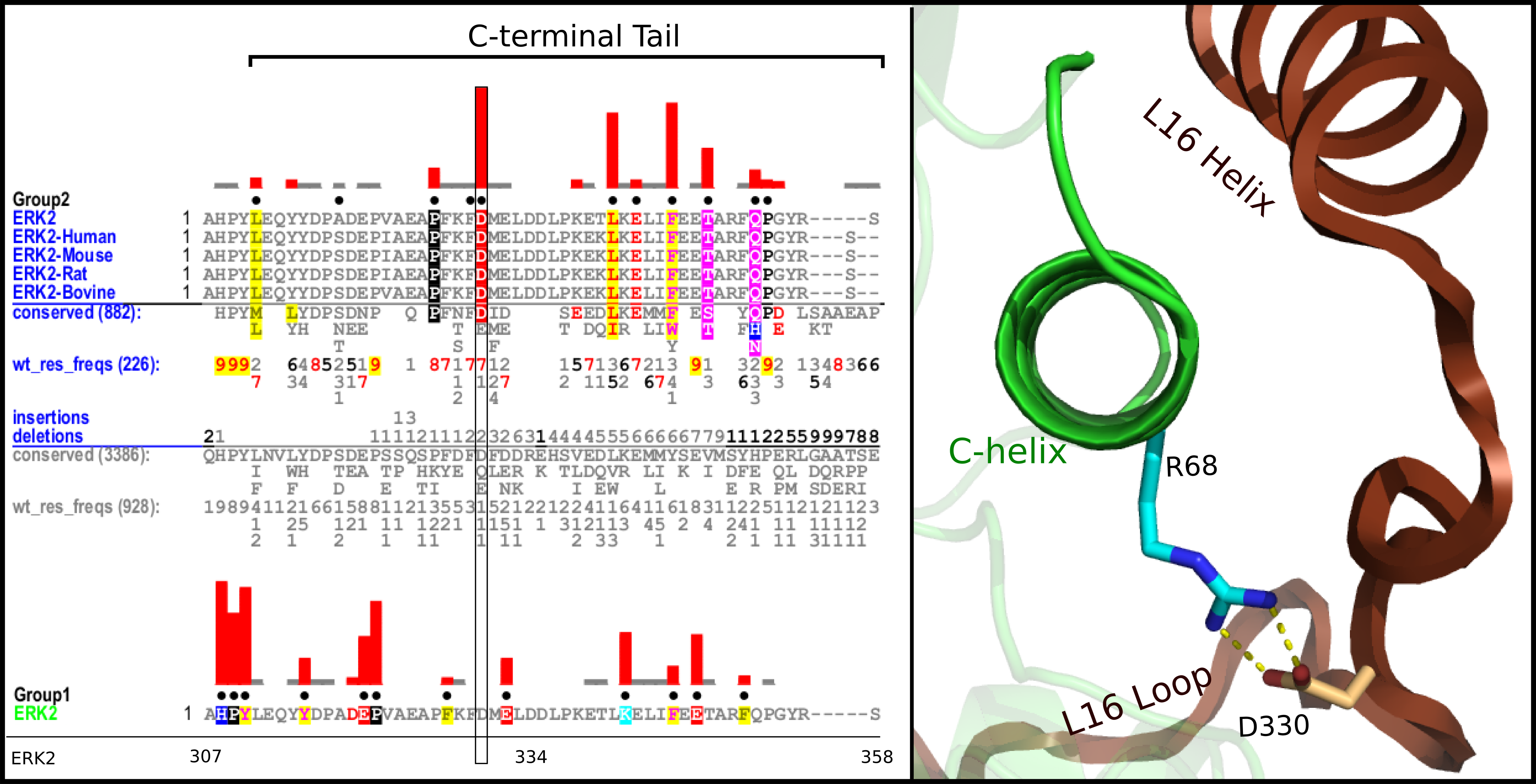

Supplement: S6 Fig — The display set shows representative ERK2 C-tail sequences. The sequence columns highlighted in black dots show the positions that most contributed to ERK1/ERK2’s shared sequence features that contributed to both families’ divergence. The highlighted column (D330ERK2) denotes the referred position discussed in the text in MD simulations section. Structural interaction of D330ERK2 observed in MD simulations is shown in the structural figure beside the alignment (PDBID:4GSB). (PNG) [file pone.0119636.s006.png]
