# Supplementary material for: Co-Conserved MAPK Features Couple D-Domain Docking Groove to Distal Allosteric Sites via the C-Terminal Flanking Tail
Source: PLoS One. 2015 Mar 23;10(3):e0119636. doi: 10.1371/journal.pone.0119636 (PMC4370755; doi:10.1371/journal.pone.0119636)
Supplement: S1 Table — (PDF) [file pone.0119636.s007.pdf]

Table S1: Hydrogen Bonding Interactions Extracted from the MD Simulations

Molecule Type	Residue i	Residue j	Regions	Occupancy	Comments
P38, peptide, C-tail	R70	D328	αC helix and L16 3/10 helix	95%	MAPK-specific salt bridge interactions
	K66	D328	αC helix and L16 3/10 helix	66%	C-capping interaction of L16 3/10 helix
	F99	K338	β4- β5 and L16 helix	12%	β4- β5 Insert interactions
	K139	P318	αE helix and L16 loop	11%	
	A144	Y323	αE helix and L16 loop	73%	MAPK-specific alanine (see Fig. 2)
	S143	P318	αE helix and L16 loop	9%	
	K76	E344	αC helix and L16 loop	90%	
	R173	Q325	A-loop and L16 loop	19%	
P38, no peptide, C-tail	R70	D328	αC helix and L16 3/10 helix	91%	MAPK-specific salt bridge interactions
	K66	D328	αC helix and L16 3/10 helix	76%	C-capping interaction of L16 3/10 helix
	F99	K338	β4- β5 and L16 helix	12%	β4- β5 Insert interactions

	K139	P318	αE helix and L16 loop	10%	
	A144	Y323	αE helix and L16 loop	6%	MAPK-specific alanine (see Fig. 2)
	S143	P318	αE helix and L16 loop	~0%	
	K76	E344	αC helix and L16 loop	92%	
	R173	Q325	A-loop and L16 loop	~0%	
ERK2, peptide, C-tail	R68	D334	αC helix and L16 3/10 helix	65%	MAPK-specific salt bridge interactions
	M96	K342	β4- β5 and L16 helix	22%	β4- β5 Insert interactions
	I93	K342	β4- β5 and L16 helix	10%	β4- β5 Insert interactions
	K71	E348	αC helix and L16 loop	77%	
	A141	F327	αE helix and L16 loop	85%	MAPK-specific alanine (see Fig. 2)
	S140	A323	αE helix and L16 loop	79%	
	D175	K328	A-loop and L16 loop	40%	
	H178	D335	A-loop and L16 loop	21%	

ERK2, no peptide, C-tail	R68	D330	αC helix and L16 3/10 helix	99%	MAPK-specific salt bridge interactions
	M96	K342	β4- β5 and L16 helix	18%	β4- β5 Insert interactions
	I93	K342	β4- β5 and L16 helix	11%	β4- β5 Insert interactions
	K71	E348	αC helix and L16 loop	67%	
	A141	F327	αE helix and L16 loop	93%	MAPK-specific alanine (see Fig. 2)
	S140	A323	αE helix and L16 loop	7%	
	D175	K328	A-loop and L16 loop	~0%	
	H178	D335	A-loop and L16 loop	~0%	
